# In-situ four-tip STM investigation of the transition from 2D to 3D charge transport in SrTiO_3_

**DOI:** 10.1038/s41598-019-38888-x

**Published:** 2019-02-21

**Authors:** Arthur Leis, Christian Rodenbücher, Krzysztof Szot, Vasily Cherepanov, F. Stefan Tautz, Bert Voigtländer

**Affiliations:** 1 Peter Grünberg Institut (PGI-3), Forschungszentrum Jülich, 52425 Jülich, Germany; 2 Jülich Aachen Research Alliance (JARA), Fundamentals of Future Information Technology, 52425 Jülich, Germany; 3 Peter Grünberg Institut (PGI-7), Forschungszentrum Jülich, 52425 Jülich, Germany; 4 Institut für Energie- und Klimaforschung (IEK-3), Forschungszentrum Jülich, 52425 Jülich, Germany; 5 A. Chełkowski Institute of Physics, University of Silesia, 40-007 Katowice, Poland

## Abstract

The electrical properties of SrTiO_3_(100) single crystals were investigated *in-situ* at different stages of thermal reduction by means of a 4-tip STM. Using the tips of the STM as electrical probes, distance-dependent four-point measurements were performed at the surface of the crystal at room temperature after reduction by thermal treatment. For annealing temperatures *T* ≤ 700 °C, charge transport is confined to a surface region <3 *μ*m below the surface. For reduction at *T* ≥ 900 °C a transition from a conducting 2D sheet with insulating bulk to a system with dominant 3D bulk conductivity is found. At an intermediate reduction temperature of *T* = 800 °C, a regime with mixed 2D/3D contributions is observed in the distance-dependent resistance measurements. Describing the depth-dependent conductivity with an analytical N-layer model, this regime of mixed 2D/3D conductivity is evaluated quantitatively under the assumption of an exponentially decaying conductivity profile, correlated with the previously observed depth-dependent dislocation density in the sample. A non-monotonous temperature dependence of the 3D conductivity in the respective conducting layer is found and possible underlying mechanisms are discussed, particularly with regard to non-intrinsic material properties depending on details of the sample preparation.

## Introduction

Strontium titanate (SrTiO_3_) has become a prototype ternary transition metal oxide due to its unique electronic properties. While being a band insulator in the stoichiometric form, it can be transformed into a metal by the generation of oxygen vacancies upon reduction^1^. Due to electronic charge compensation of positively charged vacancies by electrons, the valence state of the transition metal ion Ti is changed from +4 to +3^2^. This effect can be exploited in memristive devices based on the valence change mechanism (VCM) which holds potential for future energy-efficient data storage and neuromorphic computing^3^. Using SrTiO_3_ single crystals, it has been shown that the resistive switching can be confined to single dislocations, which act as preferential reduction sites^4^, promising that memristive data storage can be scaled down to the ultimate limit^5^. Moreover, SrTiO_3_ has been used recently to generate systems of confined metallicity such as LaAlO_3_/SrTiO_3_ interfaces^6^ showing the properties of a 2D electron gas, or high-temperature superconductors, such as FeSe grown on SrTiO_3_ ^7^. In addition, even on bare vacuum-cleaved SrTiO_3_ a 2D electron gas has been found, showing the exotic nature of the SrTiO_3_ surface^8^.

Using STM, the surface of SrTiO_3_ has been investigated extensively, showing that upon reduction, a surface transformation occurs involving reconstruction, segregation and phase transformation^9,10^. SrTiO_3_ has also become a relevant conducting substrate material when doped with Nb for the growth of functional oxide thin films as nowadays crystals with sufficiently homogeneous dopant distribution can be produced^11^.

The insulator-metal transition of a transparent conductive oxide such as SrTiO_3_ is tied to the formation of oxygen vacancies in the solid^2^. In its stoichiometric form,SrTiO_3_ is known to be an insulator with a band gap of ~3.2 eV^12,13^. As a result of thermal treatment at low oxygen partial pressure, oxygen is effused from the material, thus creating oxygen vacancies which influence the electronic structure. Upon removal of a neutral oxygen atom, two electrons are set free according to ${{\rm{O}}}_{{\rm{O}}}\to {{\rm{V}}}_{{\rm{O}}}^{\cdot \cdot }+2{e}^{-}+\frac{1}{2}{{\rm{O}}}_{2}$ (Kroeger-Vink notation) and are distributed in the *t*_2*g*_ orbitals of neighboring Ti atoms, thus lowering the oxidation state of the Ti ions. These *d*-electrons can contribute to transport as n-type charge carriers^1,14–17^. This process of formation of oxygen vacancies as a result of thermal treatment can be understood as local self-doping. Already after reduction at *T* ≈ 800 °C, SrTiO_3_ has been reported to show metallic behavior^17,18^. For higher reduction temperatures, the density of charge carriers and hence the conductivity is expected to be higher as a consequence of more oxygen vacancies being formed.

The macroscopic electronic transport properties of SrTiO_3_ can be described by the point defect chemistry model. This model takes into account the formation of oxygen vacancies and strontium vacancies as a function of the oxygen activity and temperature in thermodynamic equilibrium^2^. While this model is well-established and finds agreement with experimental investigations performed under defined oxygen activity by employing gas mixtures^19^, in this paper we focus on the initial non-equilibrium thermal reduction under ultra high vacuum conditions.

Here, we use *in-situ* distance-dependent four-point measurements in Valdes configuration to investigate the electronic transport properties on the microscale^20^, we demonstrate that the initial thermal reduction leads to the formation of a 2D conducting sheet in the surface region. With higher reduction temperature, the macroscopic specific conductivity is observed to increase by several orders of magnitude and a change of the transport regime from 2D after slight reduction to being 3D after strong reduction is detected. In the transition regime, exhibiting a mixed 2D/3D transport, the underlying conductivity profile of the reduced sample is obtained using an analytical N-layer conductance model.

## Experimental

For our measurements, we used SrTiO_3_(100) single crystal samples provided by Crystec (Berlin). All electrical measurements on the 3 × 6 × 0.5 mm^3^ sized epi-polished samples were performed *in-situ* at a pressure of $3\cdot {10}^{-10}\,{\rm{mbar}}$. As part of the investigation, the corresponding SrTiO_3_ sample under study was reduced by thermal treatment in between successive electrical measurements. For each reduction step, the sample was annealed by means of resistive current heating in the UHV environment. For this purpose, a 100 Hz AC current was applied while monitoring the resulting temperature via a pyrometer. The use of an AC current instead of a DC one prevents undesired stoichiometry polarization in the solid as a result of the applied field^21^. After slowly heating up the sample, the appropriate reduction temperature was maintained for 60 min at *p* < 10^−9^ mbar. The sample was then cooled down to room temperature carefully at a rate of ~50 °C/min, before performing the electrical measurements and the subsequent reduction step at the next higher temperature.

**Figure 1:**
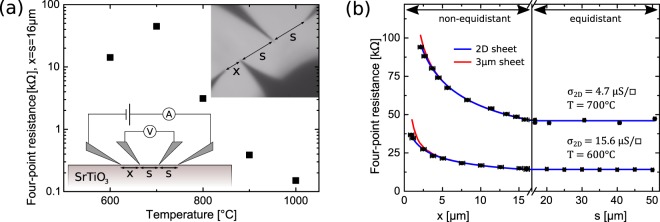
**(a)** Comparison of measured room temperature four-point resistances after annealing at different temperatures for the same arrangement of probes (i.e. *x* = *s* = 16 *μ*m). Apart from the resistance value related to annealing at *T* = 700 °C, a trend of increasing conductivity is observed for stronger reduction. The sketch corresponding to the arrangement of tips including the electrical configuration used for measuring is shown in the lower inset. The upper inset depicts an optical microscope image of an exemplary tip setup with *x* = 8 *μ*m and *s* = 16 *μ*m. **(b)** Distance-dependent four-point resistance of a SrTiO_3_(100) sample measured at room temperature after reduction at *T* = 600 °C and *T* = 700 °C. The graph includes distance-dependent measurements in both, the equidistant and the non-equidistant probe configuration, which are separated by an axis break. The blue lines represent the respective fits of the 2D resistance as a function of probe distance (Eq. 1) $x\le s=16\,\mu {\rm{m}}$ and $x=s\ge 16\,\mu {\rm{m}}$, respectively. For both sets of data, the simulated resistance curve of a sheet of 3 *μ*m thickness with corresponding constant specific conductivity is illustrated as a red line to illustrate deviations from pure 2D transport at small probe distances.

The electrical characterization was performed via four-tip STM *in-situ*^22,23^, thus avoiding oxidation processes in the specimen. With the four-tip STM allowing for free positioning of individual tips, we carried out four-point measurements for different tip distances on the SrTiO_3_(100) surface after each annealing step. The corresponding positioning of the probes was controlled visually by means of an optical microscope (upper inset of Fig. 1(a)). The lower inset of Fig. 1(a) illustrates the linear probe configuration. Two tip configurations were used. In the non-equidistant configuration, the distance *x* between one of the current-injecting and one of the voltage-probing tips is varied in between subsequent measurements, while the remaining inter-tip spacings are fixed at *s* = 16 *μ*m. In the equidistant arrangement *x* = *s*, however, all three inter-tip distances *s* are varied. Both configurations possess a specific dependence on the respective varied tip distance *x* or *s* and result for a 2D conducting sheet (2D case) and a half-infinite conducting medium (3D case) in the following equations for the four-point resistance^24,25^

1 $${R}_{2{\rm{D}}}(x,\,s)=\frac{1}{2\pi {\sigma }_{2{\rm{D}}}}[{\rm{l}}{\rm{n}}(\frac{2s}{x})+\,{\rm{l}}{\rm{n}}(\frac{s+x}{s})]$$

2 $${R}_{3{\rm{D}}}(x,\,s)=\frac{1}{2\pi {\sigma }_{3{\rm{D}}}}[\frac{1}{x}+\frac{1}{2s}-\frac{1}{s+x}].$$

The 2D and the 3D case are limiting cases. The general case is the case of a conducting layer of finite thickness *t*. Regarding the classification of transport as being 2D, one question arises: How thick does a layer have to be before deviations from the behavior in Eq. 1 are observed? For 3D transport behavior the corresponding question is: How thin can a layer be, before deviations from the 3D behavior according to Eq. 2 are observed in the measured four-point resistance? Deviations from the limiting cases for layers of finite thickness depend on the probe distance and the thickness of the conducting layer as the two relevant length scales. For inter-tip distances considerably larger than the layer thickness, transport stemming from a constant specific conductivity within the layer appears as a 2D conductivity. In case of the equidistant probe configuration, the four-probe resistance of a layer with constant specific conductivity and a thickness *t* follows the 2D function (Eq. 1) for *t/s* < 0.4, while for tip distances satisfying *t/s* > 3, the resistance is fully described by the 3D curve (Eq. 2)^26,27^. In the non-equidistant configuration, the transition between 2D and 3D conductivity is in principle dependent on both *x* and *s* as parameters, but in case of *x* < *s*, first deviations of the resistance from the 2D curve are found for *t/x* > 0.7. Simulations corresponding to these cases can be found in the Supplementary Information. As a consequence, when 2D behavior is found from the distance-dependent measurements in the non-equidistant configuration, electronic transport takes place within a depth of ≤0.7*x* below the surface.

## Results and Discussion

An overview of the experimental data is shown in Fig. 1(a) presenting the temperature dependence of the recorded four-point resistances subsequent to the reduction steps. The four-point resistances associated with a common tip arrangement ($x=s=16\,\mu {\rm{m}}$) are displayed. As a general trend, lower resistances are measured after each annealing step, apart from the case of *T* = 700 °C. After the reduction process at *T* = 600 °C, the sheet conductivity of the initially insulating sample already increases to ${\sigma }_{600{}^{\circ }{\rm{C}}}=15.6(2)\,\mu {\rm{S}}/\square$. Over the course of subsequent annealing steps, the four-point resistance drops further by two orders of magnitude (Fig. 1(a)). One distinct exception is the measured sheet resistance after reduction at *T* = 700 °C yielding ${\sigma }_{700{}^{\circ }{\rm{C}}}=4.42(3)\,\mu {\rm{S}}/\square$, which is a significant decrease compared to the prior reduction step. As this was confirmed by a very similar measurement value after repeating the annealing procedure on a different specimen, the observation is reproducible.

More detailed results of the four-probe resistance after annealing at *T* = 600 °C and *T* = 700 °C are shown in Fig. 1(b), which features the measurements with both the non-equidistant configuration with spacings $x < s=16\,\mu {\rm{m}}$ and the equidistant configuration with $x=s > 16\,\mu {\rm{m}}$. It can be seen that the data coincide very well with the 2D model in Eq. 1. The 2D character is easily recognizable from the constant resistance in the equidistant region, which is a hallmark of 2D conductivity. For the data taken in the non-equidistant configuration, a fit of Eq. 1 to the resistance confirms the 2D conductivity. The corresponding sheet conductivities have been identified as ${\sigma }_{600{}^{\circ }{\rm{C}}}=15.6(2)\,\mu {\rm{S}}/\square$ for *T* = 600 °C and ${\sigma }_{700{}^{\circ }{\rm{C}}}=4.42(3)\,\mu {\rm{S}}/\square$ for *T* = 700 °C. These conductivity values are to be understood as average quantities, which describe the electronic transport on the scale of several micrometers. When the nanometer-scale conductivity is considered, it becomes evident that the electronic transport is inhomogeneous, as explained below.

In order to evaluate how thick the 2D conducting layer close to the surface is, a simulation of the four-point resistance of a 3 *μ*m thick layer with corresponding constant specific conductivity is included in Fig. 1(b) as red lines. The simulation involves solving Laplace’s equation for a conducting layer of finite thickness on top of an insulating semi-infinite bulk^25^. In the non-equidistant configuration with *s* = 16 *μ*m, the first deviations of the simulated resistance to the pure 2D resistance occur for thicknesses *t* ≥ 3 *μ*m. This means that for *t* ≤ 3 *μ*m, charge transport in the finite layer cannot be distinguished from transport in an infinitely thin sheet in our measurement configuration. Therefore, we obtain that charge transport in the SrTiO_3_ sample at this stage of reduction is confined to *t* ≤ 3 *μ*m.

In the following we discuss why a 2D conducting layer confined to the surface region (<3 *μ*m) can be expected upon slight reduction. The initially insulating sample develops oxygen vacancies upon thermal treatment in reducing conditions. It is known from calculations^4^ that oxygen vacancies in SrTiO_3_ preferably form at dislocation cores due to a locally lower activation energy. It is also hinted by calculations^28,29^ and confirmed by high-resolution transmission electron microscopy (HRTEM) measurements^30^ that oxygen vacancies cluster linearly in the material. Furthermore, an experimental study on the dislocation density of SrTiO_3_ via local conductivity atomic force microscopy (LC-AFM) combined with etching clearly relates locally enhanced conductivity on the surface, stemming from reduction, to dislocations in the material^3^. This correlation is further supported by the investigation of reduced SrTiO_3_ bicrystals by LC-AFM showing localized conductivity close to the bicrystal boundary^31^. Since the oxygen effusion rate during reduction of SrTiO_3_ is so low that the Mott criterion is not fulfilled, as observed by mass spectrometry on a similar sample^31^, it is suggested that oxygen vacancies have to form in an ordered manner to result in a finite conductivity. Our sample under investigation was subject to mechanical polishing as part of the surface preparation procedure, which typically results in a high density of dislocations concentrated at the surface^32^. Once the reduction process is initiated, conductive paths form along the dislocations, thus constituting a network of conducting filaments close to the surface^18^. Especially for lower temperatures (*T* ≤ 700 °C), reduction is expected to occur predominantly at the surface, since the formation energy is lower for surface vacancies compared to the bulk^33^. This is in agreement with a 2D conducting layer close to the surface, which we find from our data.

Previous studies via LC-AFM have revealed inhomogeneously distributed spots with strongly enhanced nanoscale conductivities within a less conductive matrix on the sample surface as terminating points of this conductive network^5,34^. The concentration of conductive spots is reported to be of the order of 10^11^ cm^−2^. Following this estimate, an average number of about two conductive spots is covered by the approximate contact area $\pi {r}_{{\rm{tip}}}^{2}$, assuming a radius of ${r}_{{\rm{tip}}}\approx 25\,{\rm{nm}}$ for our STM tips. In fact, the contact area can be even larger than this estimate due to deformations of the sample upon contacting. Therefore, an inhomogeneous distribution of connections to the conductive network on the surface is not a concern in respect of establishing electrical contact.

**Figure 2:**
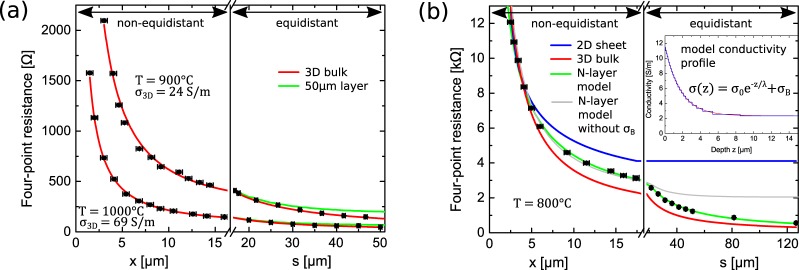
**(a)** Distance-dependent four-point resistance of a SrTiO_3_(100) sample measured at room temperature after reduction at *T* = 900 °C and *T* = 1000 °C. Data points related to a non-equidistant $x\le s=16\,\mu {\rm{m}}$ and an equidistant $x=s\ge 16\,\mu {\rm{m}}$ probe configuration are separated by an axis break at ~16 *μ*m. The red lines represent a fit of the 3D resistance (Eq. 2) to the data in both regions. The green curve represents the four-point resistance of a 50 *μ*m thick layer with the 3D conductivity obtained from the fit to the data. The deviation from pure 3D transport at large probe distances demonstrates that the conducting region extends to at least 50 *μ*m below the surface. **(b)** Four-point resistance of a SrTiO_3_(100) sample measured at room temperature after reduction at *T* = 800 °C as a function of probe distances *s* and *x* in the equidistant and non-equidistant configuration. Blue and red curves illustrate exemplary 2D and 3D resistance functions respectively. As no matching fit to all the data can be obtained with the 2D and 3D functions due to mixed contributions, the two depicted curves are fits chosen to satisfy data points for the smallest probe distances in order to emphasize deviations from the data set. The green curve represents the best fit of the N-layer model with *N* = 25 for both configuration regions. The inset depicts the corresponding conductivity profile as well as its discretization into a step function which is used as the input for the N-layer model. As seen by the disagreement to the gray curve, the N-layer model fit of a purely exponentially decaying conductivity profile fails to describe the data.

For the high annealing temperatures of *T* = 900 °C and *T* = 1000 °C, the obtained distance-dependent resistance values are found to agree with the 3D transport model as shown by the good correspondence to the red lines in Fig. 2(a), which represent a fit with Eq. 2 resulting from pure 3D conductivity. The related 3D conductivities are identified as ${\sigma }_{900{}^{\circ }{\rm{C}}}=\mathrm{23.9(5)}\,{\rm{S}}/m$ and ${\sigma }_{1000{}^{\circ }{\rm{C}}}=\mathrm{69.3(4)}\,{\rm{S}}/m$, respectively. In order to explore the sensitivity of our distance-dependent measurements of the resistance on the depth of the layer, we simulated the four-point resistance of a layer restricted to a thickness of 50 *μ*m with corresponding specific conductivities, as shown in Fig. 2(a). At this thickness, we observe the first deviations of the calculated resistance from the data, which implies that the conducting region extends beyond a depth of 50 *μ*m below the surface. We would like to note that effusion data shows that the vacancy concentration at high temperature reduction of SrTiO_3_ is still smaller than expected from Mott criterion^31^. Hence, the electrical transport which we refer to as 3D is still heterogeneous on the nanoscale following partly the hierarchical network of dislocations. The transition to 3D charge transport is explained within the framework of strong reduction of SrTiO_3_. At strongly reducing conditions, oxygen vacancies start to form even in the bulk of the sample beyond the dislocations at the surface, causing a macroscopically homogeneous 3D conductivity. Additionally, vacancies are able to diffuse from the dislocations into the bulk^35^. The resulting continuum of conductive sites is the cause for the emerging bulk conductivity eventually exceeding the surface contribution.

**Figure 3:**
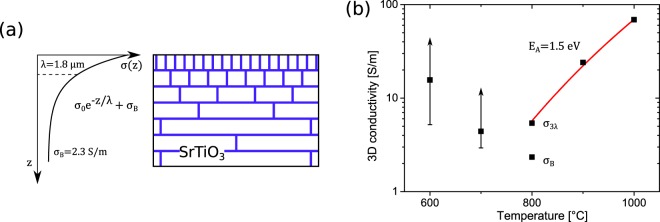
**(a)** Schematic crosssectional view of a SrTiO_3_ crystal after intermediate reduction at *T* = 800 °C and our corresponding assumed shape of the sample conductivity profile. Highly conductive regions of the sample are colored in blue while less conductive parts are uncolored. Conductivity-enhancing oxygen vacancies are introduced to the sample by means of thermal reduction. Due to the preferred segregation of oxygen vacancies at dislocation cores, the hierarchical distribution of dislocations close to the surface gives rise to a network of conducting filaments within an insulating matrix^3^. The corresponding conductivity profile *σ*(*z*) for quantitative evaluation is modeled as a saturating exponential decay. **(b)** Summary of obtained electrical conductivities of a SrTiO_3_ sample at different stages of thermal reduction. Since charge transport in SrTiO_3_ after reduction at *T* ≤ 700 °C was found to result from a 2D conductivity, the corresponding values were converted to a 3D conductivity using a finite layer thickness. Both data points were calculated assuming a 1 *μ*m-thick layer. A minimum value can be determined from the known maximum sheet thickness of 3 *μ*m as obtained from the simulation result in Fig. 1(b). As the minimum sheet thickness is not known, the upper boundary for the conductivities cannot be determined. For the case of mixed 2D/3D contributions after reduction at *T* = 800 °C, the constant bulk conductivity *σ*_B_ and the average 3D conductivity *σ*_3*λ*_ obtained from integration up to 3*λ* below the surface are presented separately.

The case of intermediate annealing at *T* = 800 °C was investigated as well, with the corresponding distance-dependent resistance data presented in Fig. 2(b). The figure also features exemplary model resistance functions for pure 2D and 3D transport. Evidently, the obtained data coincide with neither of the two and when the fit curves are chosen to agree with the data at small probe distances, the disagreement to the data is most evident. This leads to the assumption that more than a single conduction channel is involved in electronic transport in the system after reduction at *T* = 800 °C. Therefore, we attempt to describe the data using an analytical N-layer conductance model taking into account mixed 2D/3D conduction contributions^24,25^. While a simple circuit model including a 2D and a 3D channel connected in parallel could in principle be suited to describe mixed contributions, a complex distribution of charge carriers towards the bulk requires a more detailed description^25^. In order to evaluate the case of mixed 2D/3D transport quantitatively, the four-point resistance is determined for the non-trivial depth-dependent conductivity of the sample *σ*(*z*), where *z* is the spatial coordinate perpendicular to the surface. When the conductivity profile *σ*(*z*) of the sample is known, the induced electrical potential on the surface and thus the four-point resistance can be obtained analytically by solving Laplace’s equation. For this purpose, the conductivity profile *σ*(*z*) is discretized into a step-function of *N* segments, such that the resulting 2*N* + 1 boundary conditions in between can be used to find a solution for the electrical potential in the material^25^. Therefore, to obtain a resistance function ready to be fitted to our data, a model for the conductivity profile *σ*(*z*) of the sample is needed as an input. As a first approach, we assume the conductivity of the sample to be decreasing exponentially into the bulk, corresponding to the hierarchical distribution of dislocations observed by TEM in this material^3,36^,

3 $$\sigma (z)={\sigma }_{0}\cdot {e}^{-z/\lambda },$$

where *σ*_0_ and *λ* denote the fit parameters of the model. Here, we recall that *σ* is a conductivity averaged over the nanoscale conducting filaments at dislocation cores. A schematic illustration of the hierarchical distribution of conductive sites and the corresponding averaged conductivity are shown in Fig. 3(a). As can be seen from the gray curve in Fig. 2(b), a calculated fit with *N* = 25 segments matches the general trend, however the model fails to describe the recorded data at all probe distances. Especially for larger probe distances, where the measurement method is less sensitive to transport close to the surface^37^, the resistance appears to be overestimated in the model. Apparently, there is another contribution to transport that needs to be accounted for at the investigated stage of reduction. Upon inspection of quantitative investigations of the distribution of dislocations close to the surface in literature^18,32,36^, this suggestion is reinforced. Judging from the exponential decay of the dislocation density as found in literature, the distance-dependent resistance would be expected to assume a 2D behavior for even shorter probe distances than $s\approx 60\,\mu {\rm{m}}$. However, this would make the deviation from our data at large probe distances even greater. A TEM-study of a cut SrTiO_3_ surface by Wang *et al*.^36^ revealed a very steep decrease of the dislocation density in the first few micrometers below the surface. Furthermore, after only ~10 *μ*m of depth, the dislocation density was found to be saturated to a constant level. To accommodate to this, we include a bulk conductivity *σ*_B_ to the model as an additional parameter. The best fit of the extended model to the measurement data with *N* = 25 segments as seen in Fig. 2(b) as a green line is obtained for a bulk conductivity of ${\sigma }_{B}=\mathrm{2.34(1)}\,S/m$ and an initial conductivity of ${\sigma }_{0}=\mathrm{9.3(2)}\,S/m$ decaying into the bulk. The corresponding decay length is identified as $\lambda =\mathrm{1.75(4)}\,\mu {\rm{m}}$. A plot of the resulting conductivity profile as a function of depth *z* and its discretization into a step function can be seen in the inset of Fig. 2(b). The obtained decay length value, which in the context of the material is interpreted to correspond to the near-surface exponential dislocation distribution, coincides very well with the aforementioned observations found in literature. Moreover, our results are also consistent with direct observations of the conductive sites along a crosssectional cut of a SrTiO_3_ crystal via LC-AFM, where the resulting map of increased electrical conductivity is very similar to our model conductivity profile^31^.

Figure 3(b) illustrates a summary of the obtained SrTiO_3_ specific conductivities at different states of reduction from our investigation. For the sake of quantitative comparison, the respective 3D conductivities are depicted for each reduction temperature. Hence, for the data corresponding to reduction at *T* ≤ 700 °C, the obtained 2D conductivity values were converted to 3D conductivities assuming a finite layer thickness of *t* = 1 *μ*m. From our simulation results presented in Fig. 1(b), we know that charge transport at the corresponding stage of reduction is confined to *t* < 3 *μ*m below the surface, which enables us to determine a lower boundary for the converted 3D conductivity (as indicated in Fig. 3(b)). As the minimum thickness of the conducting layer is unknown, no upper boundary can be determined. In the case of mixed 2D/3D transport contributions after reduction at *T* = 800 °C, the constant 3D bulk conductivity *σ*_B_ and the average conductivity in the region of the exponential decay *σ*_3*λ*_ are plotted separately. At higher annealing temperatures, the determined 3D conductivity increases in agreement with the assumption that with prolonged reduction, more oxygen vacancies are induced. Here, it has to be noted that the maximum analysis depth of our method is ~100 *μ*m. Hence, the interpretation of a 3D conductivity refers to a depth of 100 *μ*m.

For the two lowest annealing temperatures (600 °C and 700 °C) the trend is opposite: higher 3D conductivity at lower temperatures. For the conversion from the 2D conductivity to the 3D conductivity, the same thickness of the 2D sheet was used (1 μm for the data point and 3 μm for the lower bound of the conductivity). If, more realistically, a thinner sheet thickness is used for the lower annealing temperature, the trend of a higher 3D conductivity at lower temperatures will be even stronger. The interpretation of the obtained conductivity values after low temperature reduction is not straightforward. The unexpected decrease in conductivity after reduction at 700 °C is not observed in macroscopic resistance measurements, which is shown in the Supplementary Information for a similar sample. Therefore, we attribute this observation to be due to surface properties of our sample. Since the preparation of the sample was done by the manufacturer in ambient conditions, contaminations on the surface can have an effect on our low temperature data. Therefore, the obtained conductivities after reduction at low temperatures are not to be regarded as an intrinsic material property.

The decrease of conductivity at 700 °C might stem from a rearrangement or annihilation of dislocations upon annealing and the subsequent redistribution of oxygen vacancies. Also, the thermal activation of the Si heater serving as a getter material may contribute to this trend. Furthermore, carbon contaminants at the surface can have a significant effect. From XPS measurements of adsorbates on a similar sample, carbonates from contaminations on the SrTiO_3_ surface are found dissolve at the surface at 700 °C according to ${{\rm{CO}}}_{2}\to {\rm{CO}}+\frac{1}{2}{{\rm{O}}}_{2}$, leaving behind oxygen that can effuse or be incorporated in the crystal^38^. The effusion is observed by complementary mass spectrometry data showing an increased effusion rate of oxygen at 700 °C compared to 800 °C^31^. With the remaining oxygen incorporated in the sample, it is expected to see a decrease in conductivity in surface-sensitive measurements.

At higher annealing temperatures the above effect is exceeded by the formation of oxygen vacancies beyond the dislocations under the strongly reducing conditions. This is in accord with the observed Arrhenius behavior as seen in Fig. 3(b) in the temperature range between 800 °C and 1000 °C, as it is expected for a thermally activated process like the formation of oxygen vacancies. We find the corresponding activation energy to be ${E}_{A}=\mathrm{1.5(2)}\,{\rm{eV}}$. In total two opposing processes, one decreasing the conductivity with temperature and another, leading to an increase of the conductivity with temperature, result in the observed non-monotonous behavior of the conductivity as function of the annealing temperature.

## Conclusion

We have investigated the charge transport characteristics of an epi-polished SrTiO_3_(100) sample after successive stages of thermal reduction in vacuum. By using a four-tip STM, we were able to perform distance-dependent four-point resistance measurements on the sample surface *in-situ*, thus preventing a change of the conductivity e.g. due to re-oxidation at ambient conditions. The measurements result in depth-dependent information about the average conductivity of the SrTiO_3_ sample. Our results clearly show a transition from 2D to 3D transport with stronger reduction, which can be explained by a dislocation-based interpretation of the thermal reduction process. At lower annealing temperatures, oxygen vacancies preferably introduced at dislocation cores follow the density of dislocations concentrated to the surface, thus constituting a network of conducting filaments resulting in a 2D conductance. Upon strong reduction, a 3D conductivity is observed, showing that oxygen vacancies contributing to the electronic transport have also been generated in deeper parts of the crystal and in the matrix in between the dislocations.

Furthermore, we quantitatively analyzed the transport characteristics in the intermediate reduction stage containing mixed 2D/3D contributions. With the dislocation profile of the sample being known from TEM results^36^, we were able to obtain a conductivity profile fully describing the recorded resistance data.

## Supplementary information

Supplementary Information for In-situ four-tip STM investigation of the transition from 2D to 3D charge transport in SrTiO3

## Data Availability

Data within the manuscript and its Supplementary Information is available from the corresponding author upon reasonable request.
