# Supplementary material for: In-situ four-tip STM investigation of the transition from 2D to 3D charge transport in SrTiO3
Source: Sci Rep. 2019 Feb 21;9:2476. doi: 10.1038/s41598-019-38888-x (PMC6384903; doi:10.1038/s41598-019-38888-x)
Supplement: Supplementary file 1 — Supplementary Information for In-situ four-tip STM investigation of the transition from 2D to 3D charge transport in SrTiO3 [file 41598_2019_38888_MOESM1_ESM.pdf]

Supplementary Information for

In-situ four-tip STM investigation of the transition from 2D to 3D charge transport in $SrTiO_3$

Arthur Leis, Christian Rodenbücher, Krzysztof Szot, Vasily Cherepanov, F. Stefan Tautz, Bert Voigtländer

1. Deviation of the four-point resistance of conducting sheets with finite thickness from ideal 2D transport

In case of an infinitesimally thin conducting sheet, current injected from tips spreads cylindrically from the current-injecting electrodes and charge transport only takes place in-plane. The corresponding four-point resistance can be calculated according to Eq. 1 of the main text.

How much the measured distance-dependent four-point resistance deviates from the limiting case of 2D behavior depends on the probe distance in relation to the thickness of the conducting layer. In case of a small sample thickness t compared to the probe distances x and s, the current propagation is practically in-plane on the scale of the distance of the voltage-probing tips and thus, the four-point resistance of the thin finite layer follows the 2D resistance behavior. The corresponding observed 2D conductance of a sample with constant 3D specific conductivity σ_{3D} is related to the 3D specific conductivity via $\sigma_{\mathrm{2D}} = \sigma_{\mathrm{3D}} t$.

In order to estimate for which sheet thickness a deviation from the 2D behavior occurs (for a particular measurement configuration), the four-point resistance of a conducting layer of finite thickness has to be calculated. We follow the procedure outlined in Just et al.[1]. For our calculations, we consider a system of two layers: one infinite sheet with thickness t and constant 3D conductivity σ_{3D} on top of a half-infinite insulating bulk (Fig. S1). Solving Laplace's equation and obeying the boundary conditions given by the continuity of the electrical potential and the current density between the two layers, the four-point resistance on the surface is obtained from the analytical expression[1]

$$R_{4p}(x,s) = -\frac{1}{I}\int_0^\infty [a_1(k)+a_2(k)]\cdot [J_0(ks) - J_0(2ks) + J_0(kx) - J_0(k(s+x)]\, dk \quad (1)$$

where J_0 denotes the Bessel function of the first kind, while a_1 and a_2 are parameters containing the conductivity and the thickness of the infinite sheet.

The resulting four-point resistance of the two-layer system relative to the 2D resistance of an infinitely thin sheet (Eq. 1 of the main text) is shown in Fig. S2 for various ratios of sheet thickness t to probe distance s. As can be seen from the figure, the four-point resistance of the finite sheet is equal to the 2D resistance in Eq. 1 in the main text in the limit of large probe distances $\mathrm{x/t} \gg 1$. In the considered case of the non-equidistant probe-configuration ($x < s$), the probe distance x/t, at which deviations from the 2D limit can be detected for a layer of finite thickness, is found to depend on the probe distance s. However, we can say that in the case of $x < s$, for sheets that are thinner than $t \lesssim$ 0.7x, 3D transport within the finite layer cannot be distinguished from ideal 2D transport by means of four-point resistance measurements, i.e. R_{4p} according to Eq. 1 is equal to R_{2D} from Eq. 1 of the main text. With that in mind, we can say that whenever 2D behavior of the four-point resistance is found in our experiment, charge transport takes place within a depth of $t \lesssim$ 0.7x below the surface.

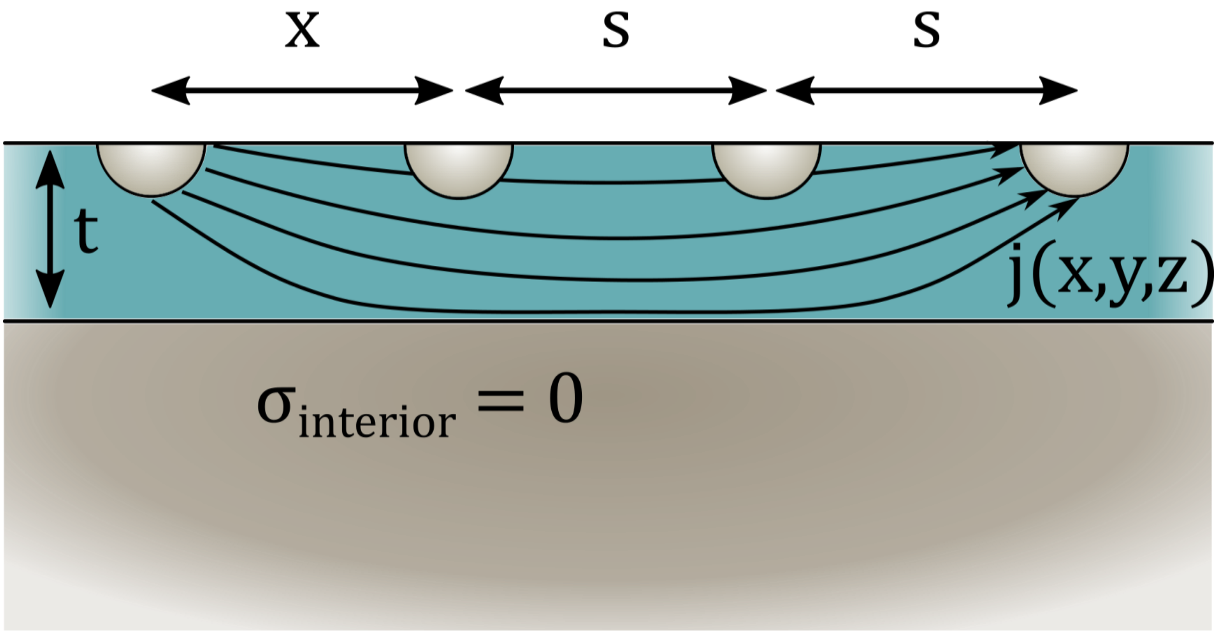

Figure S1. Schematic view of the two-layer system considered in our simulation. Current induced by the outer tips can propagate in the conducting sheet of thickness t in a different way than it would be the case for pure 2D transport. The insulating layer below prevents any current flow in its interior. The related boundary value problem is solved, yielding the four-point resistance of the two-layer system with the probe distances (x and s) and the sheet thickness t as parameters.

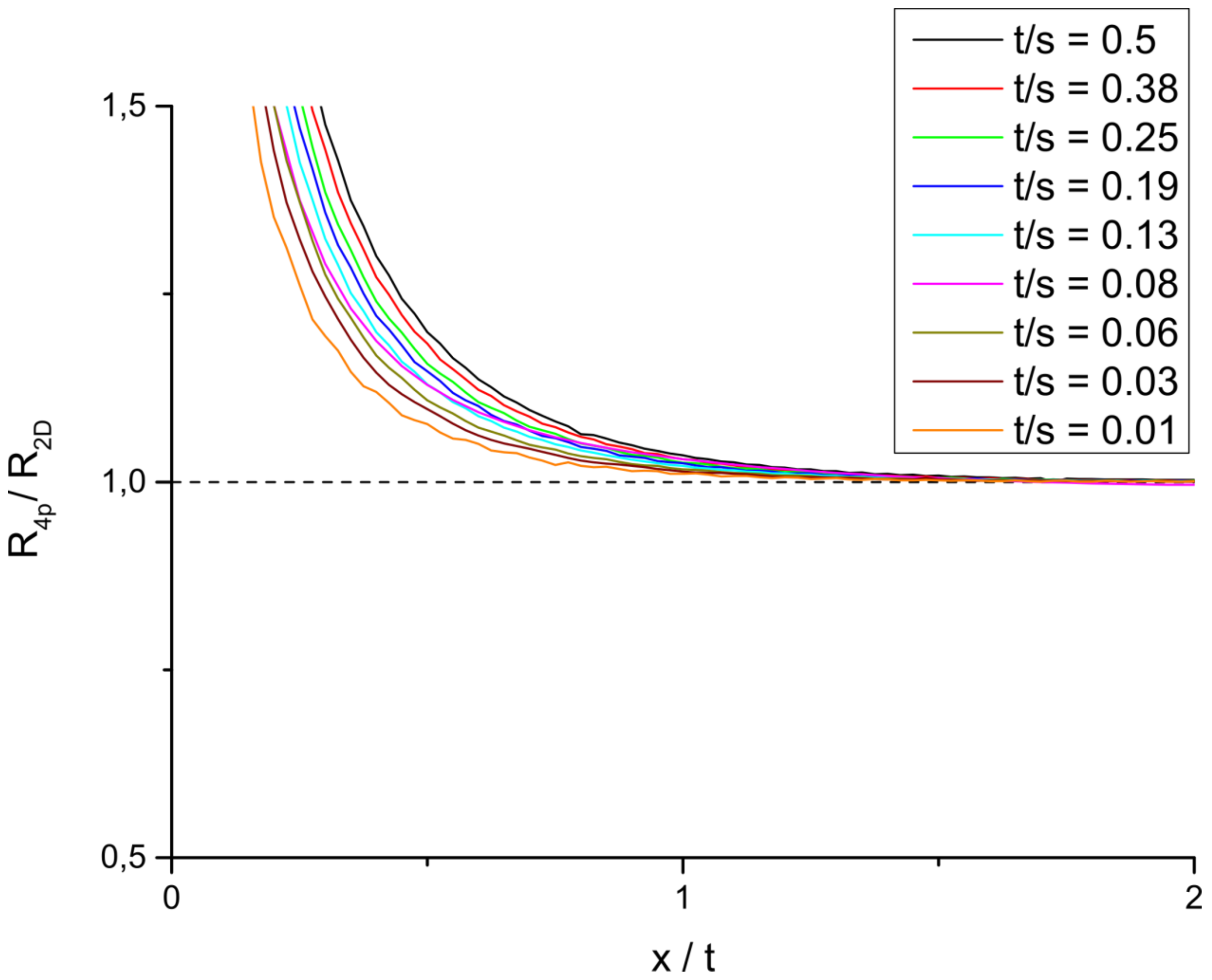

Figure S2. Four-point resistance of a conducting sheet of thickness t on top of an insulating bulk, as obtained from the evaluation of the analytical expression in Eq. 1, in relation to the 2D resistance of an infinitely thin sheet with corresponding conductance $\sigma_{3D} \cdot t$. The unitless resistance is plotted as a function of normalized tip distance x/t. Deviations of the obtained finite-sheet resistance from the 2D resistance are observed at $x/t \lesssim 1.3$.

2. Macroscopic four-probe measurements

In order to investigate the electrical properties of the sample during thermal reduction, macroscopic four-probe measurements were conducted. An as-received $SrTiO_3$ single crystal ($10 \times 3 \times 0.5$ mm^3) was contacted with four electrodes that were fabricated by applying Pt paste. Subsequently, Pt wires (0.3 mm in diameter) were wrapped around the pasted electrodes. The outer electrodes were connected to a voltage source and the inner electrodes were connected to sensitive electrometers. An AC voltage of 4 mV at a frequency of 171 Hz was used. This was done to prevent stoichiometry polarization within the sample that is known to occur when using DC voltage. The current through the outer electrodes and the potential at the inner electrodes were recorded using lock-in technique. The measurements were performed in

situ under vacuum conditions ($p < 10^{-6}$ mbar). The sample was placed inside a quartz tube that was heated by an external tube furnace allowing for repeated measurements at different temperatures without changing the position of the electrodes. Here, we would like to note that due to the higher absolute pressure and the use of an external heater instead of a Si heater, as was used during the 4-tip STM measurements, the local oxygen partial pressure close to the sample was slightly higher but we still consider the experimental conditions as comparable.

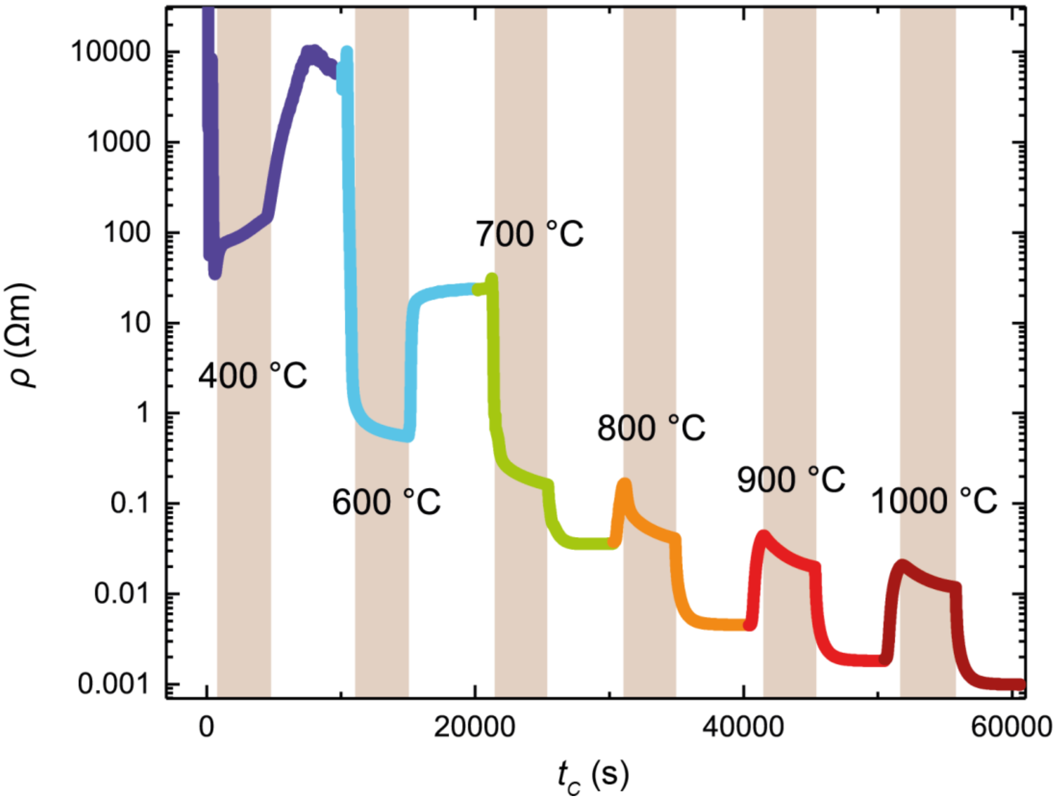

Figure S3. Resistivity of the $SrTiO_3$ crystal during annealing at different temperatures and subsequent cooling to room temperature.

In analogy to the 4-tip STM measurements the sample was subsequently heated to different temperatures of 400°C, 600°C, 700°C, 800°C, 900°C and 1000°C. The heating rate was approx. 4 °C/min. After the respective temperature was reached, it was held for 1 h before the sample was cooled down to room temperature again. Fig. S3 displays the resistivity of the sample measured during the treatment. Here t_c denotes the corrected time meaning that breaks at room temperature between the measurements are not shown. The beige-coloured areas denote the annealing periods at the respective temperatures. It can be seen that with increasing temperature, the resistivity of the sample is decreasing as function of the reduction. This is illustrated in Fig. S4 showing the resistivity values measured at room temperature after each

reduction step at the respective annealing temperature T_A. A decrease of several orders of magnitude is observed. Additionally, regarding the cooling curves in Fig. S3, it can be seen that during cooling from 400°C and 600°C, the resistivity increased again while at all temperatures above 700°C the resistivity decreased during cooling. This indicates that an insulator-metal transition occurred during annealing at 700°C.

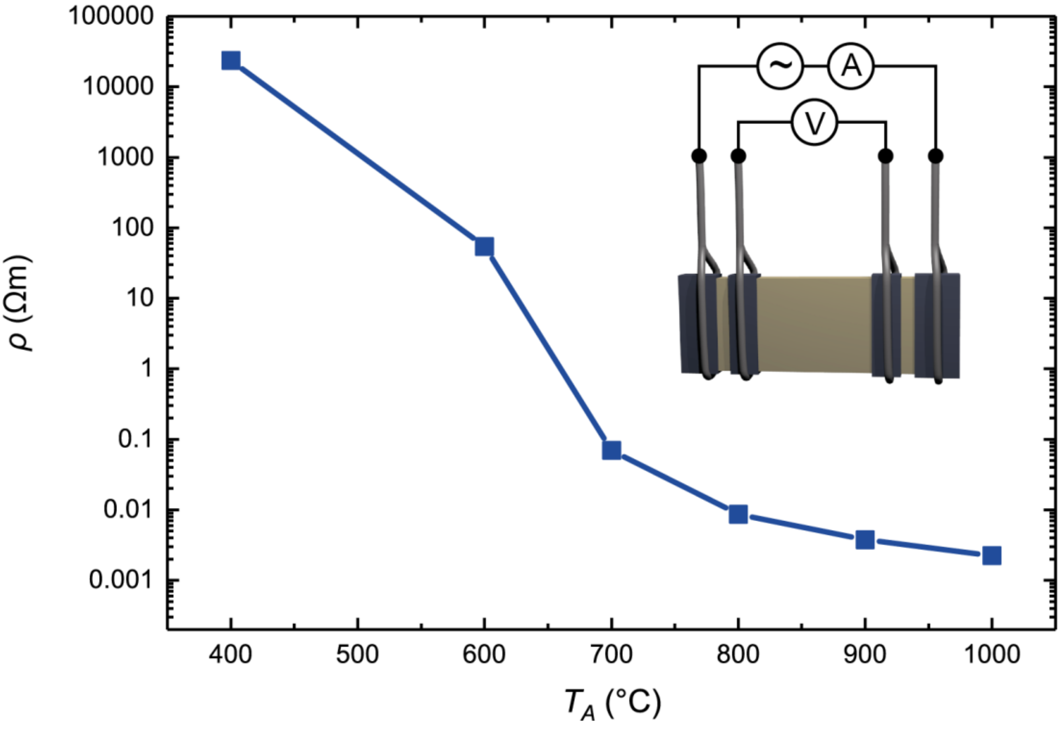

Figure S4. Room temperature resistivity of the $SrTiO_3$ crystal measured after each annealing step. The inset depicts the geometry of the setup.

References

1. Just, S., Soltner, H., Korte, S., Cherepanov, V. & Voigtländer, B. Surface conductivity of Si(100) and Ge(100) surfaces determined from four-point transport measurements using an analytical *N*-layer conductance model. *Phys. Rev. B* **95**, 075310 (2017).
